# Prompt-Sensitive Decision Behavior of Large Language Models in Intensive Care Unit Mortality Prediction for Spontaneous Intracerebral Hemorrhage: Comparative Benchmarking Study

**DOI:** 10.2196/29701

**Published:** 2026-07-08

**Authors:** Jinn-Rung Kuo, Guan-Yu Chen, Xiao-Han Vivian Yap, Chao-Chien Li, Yung-De Kuo, Chung-Feng Liu

**Affiliations:** 1 School of Medicine, College of Medicine, National Sun Yat-Sen University, Kaohsiung, Taiwan; 2 Department of Medical Research, Chi Mei Medical Center, Tainan, Taiwan; 3 Department of Neurosurgery, Chi Mei Medical Center, 901 Chung Hwa Road, Yung Kang DistrictTainan, Taiwan, 886 6-281-2811 ext. 5

**Keywords:** intracerebral hemorrhage, machine learning, large language models, structured clinical prediction, prompt-sensitive behavior, explainable artificial intelligence, Shapley Additive Explanations, SHAP

## Abstract

**Background:**

Large language models (LLMs) are increasingly being explored for clinical decision support. However, whether inference-only LLM outputs can be interpreted as reliable quantitative risk estimates in structured clinical prediction remains unclear.

**Objective:**

This study aimed to evaluate the predictive performance and decision-making behavior of inference-only LLMs in a structured clinical prediction task and compare their outputs with those of an outcome-trained machine learning model.

**Methods:**

We conducted a controlled benchmarking study using identical structured clinical inputs from patients admitted to intensive care units with spontaneous intracerebral hemorrhage. An outcome-trained extreme gradient boosting model was compared with predictions generated by a general-purpose LLM using four prompting strategies: zero-shot, few-shot, chain-of-thought, and combined few-shot plus chain-of-thought prompting. Performance was evaluated using discrimination metrics, threshold-dependent classification behavior, and concordance between Shapley Additive Explanations–derived feature importance rankings and LLM-derived feature prioritization. The independent testing cohort included 435 patients, of whom 86 (19.7%) experienced in-hospital mortality.

**Results:**

The outcome-trained machine learning model demonstrated superior discriminative performance compared with all LLM-based approaches. LLM predictions achieved moderate discrimination but exhibited substantial variability in threshold-dependent classification behavior across prompting strategies. At a fixed probability threshold of 0.5, LLM approaches consistently demonstrated high sensitivity and lower specificity, whereas operating characteristics varied considerably when thresholds were optimized using the Youden index. The optimal thresholds for LLM-based approaches ranged from 0.74 to 0.88, compared with 0.1555 for the extreme gradient boosting model. Concordance between Shapley Additive Explanations–derived attribution and LLM-derived feature prioritization was modest, suggesting only partial alignment between empirically learned predictor structure and language-based reasoning patterns.

**Conclusions:**

In this structured clinical prediction setting, inference-only LLM outputs demonstrated prompt-sensitive decision behavior despite moderate discriminative performance. These findings suggest that LLM-generated probability outputs should be interpreted cautiously when used for quantitative clinical risk estimation. A complementary framework integrating outcome-trained predictive models with LLM-assisted reasoning may provide a more reliable direction for future clinical decision support systems.

## Introduction

Spontaneous intracerebral hemorrhage (SICH) is one of the most devastating forms of stroke and remains associated with high early mortality and substantial long-term disability [1,2]. Patients admitted to the intensive care unit (ICU) with SICH often present with severe neurological impairment and rapid physiological deterioration, making early mortality risk estimation both clinically important and methodologically challenging [3,4].

Traditional prognostic approaches for SICH—including clinical scoring systems and regression-based models—typically rely on a limited set of neurological or radiographic variables and demonstrate heterogeneous performance across patient cohorts [1,5]. In recent years, machine learning methods have increasingly been applied to clinical prediction tasks using structured electronic health record data. Tree-based ensemble algorithms, such as extreme gradient boosting (XGBoost), can capture nonlinear relationships and complex feature interactions, enabling improved predictive discrimination in clinical risk modeling [6,7]. Deep learning approaches have further expanded the scope of data-driven clinical prediction [8]. In particular, recent work has demonstrated that high-performance machine learning models can be developed for mortality prediction in patients with SICH using structured ICU data [9]. Despite these advances, concerns remain regarding interpretability, calibration, and reliability when predictive models are deployed in high-stakes clinical environments [10,11].

More recently, large language models (LLMs) and foundation models have attracted substantial attention in medicine because of their ability to encode broad biomedical knowledge and generate structured clinical reasoning [12-14]. The integration of artificial intelligence (AI) with clinical medicine has been proposed as a paradigm shift toward high-performance medicine [15]. However, whether inference-only LLMs can provide reliable probability estimates for structured clinical prediction tasks remains uncertain.

Importantly, methodological differences distinguish outcome-trained predictive models from inference-only language models. Machine learning models are explicitly optimized using outcome-labeled data and evaluated using established statistical frameworks for discrimination, calibration, and validation [11,16]. In contrast, most LLMs are deployed without task-specific training or outcome calibration, generating probability-like outputs through language-mediated reasoning processes that may be sensitive to prompting strategies and contextual framing [10,17]. Emerging evidence suggests that such systems may produce clinically coherent outputs that do not necessarily correspond to empirically optimized predictive structures [17]. Recent studies have further highlighted limitations in the reliability and consistency of clinical reasoning in LLMs [18].

A critical but underexplored question is how probability-like outputs generated by inference-only LLMs should be interpreted in structured clinical prediction tasks. While prior studies have primarily evaluated LLM performance using discrimination metrics or task accuracy, less attention has been given to threshold-dependent decision behavior and the relationship between language-based reasoning patterns and empirically learned predictive structures when these systems are applied to structured clinical data.

In parallel, another unresolved issue concerns the relationship between statistically derived feature attribution and language-based clinical reasoning. Explainability frameworks such as Shapley Additive Explanations (SHAP) enable the quantification of feature contributions within trained predictive models by estimating marginal effects grounded in observed data [19,20]. In contrast, LLM-generated explanations typically reflect narrative reasoning processes derived from generalized medical knowledge rather than empirically optimized predictive structures [17].

To address these gaps, we propose a conceptual framework distinguishing outcome-optimized predictive models from language-based inference systems. The conceptual framework underlying this study is illustrated in Figure S1 in Multimedia Appendix 1. In this framework, machine learning models are optimized for predictive faithfulness, defined as alignment with empirically observed outcome patterns, whereas inference-only LLMs generate outputs through knowledge-driven reasoning processes that are not explicitly optimized for quantitative probability estimation.

Guided by this framework, this study builds upon a previously developed outcome-trained machine learning model [9] to enable a controlled comparison with inference-only LLM systems. Specifically, we aimed to evaluate the performance and decision behavior of inference-only LLMs when applied to structured clinical prediction tasks using identical clinical inputs. We compared predictive discrimination, threshold-dependent classification behavior, and feature attribution patterns between an outcome-trained machine learning model and an LLM under multiple prompting strategies.

We hypothesized that inference-only LLMs would demonstrate different operational characteristics from an outcome-trained machine learning model, including greater sensitivity to prompting conditions and weaker alignment between language-based feature prioritization and empirically derived predictor importance.

## Methods

### Ethical Considerations

This study was approved by the Institutional Review Board of Chi Mei Medical Center (IRB 11407‐008). All procedures were conducted in accordance with the ethical standards of the institutional research committee and the principles of the Declaration of Helsinki. Given the retrospective study design and the use of deidentified clinical data, the requirement for informed consent was waived by the Institutional Review Board.

### Study Design

A retrospective benchmarking study was conducted to compare an outcome-optimized machine learning model with an inference-only LLM for predicting in-hospital mortality in patients with SICH. The study design and reporting approach were informed by established methodological principles for clinical prediction model evaluation, model interpretability, and transparent reporting [16,20,21]. Because this study represents a comparative benchmarking analysis rather than a conventional prediction model development or validation study, no single existing reporting guideline fully captures all aspects of the study design.

Guided by the conceptual framework illustrated in Figure S1 in Multimedia Appendix 1, this study was designed to compare 2 distinct paradigms of clinical AI: outcome-optimized predictive modeling and inference-only language-based reasoning. To ensure a controlled comparison, both systems were evaluated using identical structured clinical inputs and an independent testing cohort. In addition to predictive discrimination, we assessed threshold-dependent classification behavior and concordance in feature prioritization to examine the extent to which language-based reasoning patterns aligned with empirically derived predictive structures.

### Data Source and Study Population

Data were obtained from a previously described multicenter ICU cohort of patients with SICH [9]. The dataset included 1451 adult patients admitted to 3 hospitals between January 1, 2016, and December 31, 2021.

The dataset was randomly divided into a training cohort of 1016 patients and an independent testing cohort of 435 patients, corresponding to a 7:3 split ratio. The index time point was defined as ICU admission, and the primary outcome was all-cause in-hospital mortality.

Each patient record included 18 structured clinical variables collected at ICU admission, representing neurological status, physiological support, and baseline comorbid conditions. These variables were selected based on prior clinical modeling studies and their availability across all participating hospitals. Variables included pupil light reflex, pupil size, Glasgow Coma Scale (GCS) components (eye-opening score, verbal score, and motor score), vasopressor use, fraction of inspired oxygen (FiO_₂_), external ventricular drainage, extremity muscle power, diabetes mellitus, hypertension, kidney disease, and surgical intervention.

All variables were extracted from electronic health records and anonymized prior to analysis. The benchmark XGBoost model evaluated in this study was based on the authors’ previously published mortality prediction framework, which was developed using the same ICU cohort and training dataset [9].

### Machine Learning Model

The reference machine learning model consisted of an XGBoost-based predictive model that was previously developed using the same dataset [9]. The model was previously trained using the same training cohort described in the original study. Model performance was assessed using accuracy, sensitivity, specificity, and the area under the receiver operating characteristic curve (AUC). The same testing dataset was subsequently used for all LLM-based inference strategies.

XGBoost is a gradient-boosted decision tree algorithm widely used for clinical prediction tasks because of its ability to capture nonlinear relationships and complex feature interactions [6].

To ensure methodological comparability, the previously trained model was applied without retraining or modification. All hyperparameters and the predefined classification threshold were preserved, as reported in the original study. Model performance was evaluated using the independent testing dataset.

### LLM Framework: Model Deployment

The LLM evaluated in this study was ChatGPT, accessed through the ChatGPT web interface in March 2026. According to OpenAI’s public documentation available during the inference period, this model option corresponded to GPT-5.3 Instant. The model was deployed strictly in inference-only mode without parameter fine-tuning, gradient updating, or external knowledge retrieval. Because the web interface did not expose a checkpoint identifier, hidden model subversion, or complete inference configuration, the exact checkpoint-level model version could not be further specified. Therefore, the LLM results should be interpreted as observations from the GPT-5.3 Instant web-interface model used during the inference period, rather than from a version-locked API model.

To ensure a controlled comparison, the LLM received exactly the same 18 structured clinical variables used in the machine learning model and was not provided with additional contextual information beyond the prompting instructions described in Multimedia Appendix 2.

All model inferences were performed using identical prompts and structured input formatting across runs to reduce variability and improve reproducibility. All prompt templates, structured input formats, and inference instructions are provided in Multimedia Appendix 2.

Our objective was not to optimize LLM performance through task-specific fine-tuning, but rather to evaluate how a general-purpose, inference-only LLM generates probability-like outputs when applied to structured clinical prediction tasks without outcome-specific training.

### Prompting Strategies

The LLM was evaluated under 4 prompting strategies. Chain-of-thought prompting has been shown to improve structured reasoning performance in LLMs [22,23].

1. Zero-shot prompting, in which predictions are generated without exemplar cases.
2. Few-shot prompting, in which exemplar mortality cases from the training dataset are provided, was used. The few-shot prompting strategy used outcome-labeled mortality cases selected from the training cohort as exemplars. Only mortality cases were included in the exemplar set. This approach was intended to provide representative examples of high-risk clinical presentations while maintaining a manageable prompt length within the constraints of the deployment environment. Although the few-shot condition incorporated outcome-labeled exemplars through in-context prompting, no parameter updating or model fine-tuning was performed.
3. Few-shot prompting with chain-of-thought reasoning combines exemplar guidance with structured reasoning instructions.
4. Chain-of-thought prompting only, in which structured reasoning prompts are applied without exemplar cases.

For each patient record, the LLM generated a continuous mortality probability ranging from 0 to 1.

### Prediction Output and Threshold Definition

Formal calibration analyses were not performed in this exploratory benchmarking study. Accordingly, model outputs were interpreted as relative probability-like scores rather than calibrated risk estimates. Predicted probabilities were recorded to 2 decimal places as returned by the model interface. The model was instructed to output a numeric probability between 0 and 1, representing the relative in-hospital mortality likelihood

Two classification thresholds were evaluated: (1) a fixed probability threshold of 0.5 and (2) a balanced operating threshold derived using the Youden Index [24].

### LLM Inference Output Quality Control

The prompt template prespecified the required output format, including one numeric in-hospital mortality probability between 0.00 and 1.00 for each patient, CSV-formatted output, probabilities standardized to 2 decimal places, preservation of the original row order and original columns, and the addition of only the “AI” column. The instruction to process datasets in batches of up to 500 patients was used as an operational batching rule rather than as a formal token-limit estimate.

For each prompting strategy, inference was conducted in a newly opened, independent ChatGPT web-interface session to minimize context carryover and potential memory-related bias across experiments. The complete prompt template and structured input instructions were reentered at the beginning of each session. Because the testing cohort contained 435 patients, the prespecified batching rule was not triggered, and each prompting strategy was submitted as a single batch.

After each inference run, the researchers compared the returned outputs with the original testing dataset before conducting statistical analysis. The quality control checks included verification of the total row count, preservation of the original row order, presence of exactly one “AI” probability value for each patient, numeric-format checking, and range checking to confirm that all probability values were between 0.00 and 1.00. Missing, duplicated, refused, malformed, nonnumeric, or out-of-range outputs were predefined as noncompliant outputs requiring manual review and reprocessing using the same prompt and structured input format.

### Model Performance Evaluation

Model discrimination was assessed using the AUC with corresponding 95% CIs.

Pairwise comparisons between the machine learning model and each LLM configuration were performed using the DeLong test for correlated receiver operating characteristic curves [25]. A 2-sided *P* value of less than .05 was considered statistically significant.

All statistical analyses were performed using SPSS for Windows (version 26; SPSS Inc). Receiver operating characteristic analysis and DeLong tests were implemented using standard statistical procedures.

### Explainability Analysis

Feature importance for the XGBoost model was evaluated using SHAP, a game-theoretic framework that quantifies the contribution of individual predictors to model predictions [19,20]. SHAP was selected because it provides theoretically grounded feature attribution based on Shapley values and has become a widely adopted framework for interpreting tree-based predictive models.

Global feature importance was calculated using the mean absolute SHAP value across the testing cohort.

To evaluate language-based reasoning patterns, the LLM was additionally instructed to rank the relative importance of the 18 clinical variables and assign ordinal weights ranging from 1 to 10.

These rankings were intended to reflect qualitative reasoning patterns under different prompting conditions rather than formal feature attribution comparable to SHAP-derived importance values.

### Concordance Analysis

To assess the relationship between statistically derived feature attribution and language-based feature prioritization, the Spearman rank correlation coefficients (ρ) were calculated between SHAP-derived feature importance and LLM-derived rankings. Spearman correlation was selected because LLM feature weights represent ordinal rankings rather than continuous variables.

## Results

### Study Cohort

The independent testing cohort consisted of 435 adult ICU patients with SICH, consistent with the previously described dataset [9]. All patients had complete outcome data for in-hospital mortality and complete values for the 18 structured clinical variables used as model inputs.

The prevalence of in-hospital mortality in the testing cohort was 19.7% (86/435 patients).

The identical testing dataset was applied to both the previously developed XGBoost model and all LLM-based inference strategies to ensure methodological comparability.

### LLM Inference Output Quality Control Results

Across all 4 prompting strategies, the returned outputs contained 435 rows and were successfully matched to the original testing dataset. The original row order was preserved, and each patient received exactly one numeric probability value in the “AI” column. All probability values were within the prespecified range of 0.00 to 1.00. No missing, duplicated, refused, malformed, nonnumeric, or out-of-range outputs were identified; therefore, no LLM-generated output was excluded, manually corrected, or reprocessed.

### Model Performance at a Fixed Probability Threshold (*P*=.5)

At the predefined probability threshold of 0.5, the outcome-optimized XGBoost model achieved an AUC of 0.913 (95% CI 0.882‐0.944), representing the highest discriminative performance among the evaluated models (Table 1). At this threshold, the XGBoost model achieved an accuracy of 0.878, a sensitivity of 0.581, and a specificity of 0.951.

**Table 1.** Model performance using a fixed probability threshold (*P*=.5).

Models head	Threshold	Accuracy	Sensitivity	Specificity	AUC^a^ (95% CI)	*P* value
XGBoost^b^	0.5	0.878	0.581	0.951	0.913 (0.882‐0.944)	—^c^
Zero-shot Learning	0.5	0.64	0.965	0.56	0.874 (0.837‐0.911)	<.001
Few-shot Learning	0.5	0.5	0.988	0.38	0.861 (0.82‐0.901)	<.001
Few-shot+ ^d^CoT	0.5	0.716	0.895	0.671	0.864 (0.823‐0.904)	<.001
CoT	0.5	0.697	0.895	0.649	0.868 (0.83‐0.906)	<.001

a AUC: area under the receiver operating characteristic curve.

b XGBoost: extreme gradient boosting.

c Not applicable.

d CoT: chain-of-thought.

In comparison, the zero-shot prompting configuration achieved an AUC of 0.874 (95% CI 0.837‐0.911) and demonstrated an accuracy of 0.640, a sensitivity of 0.965, and a specificity of 0.560.

The few-shot prompting configuration achieved an AUC of 0.861 (95% CI 0.820‐0.901) and demonstrated an accuracy of 0.500, a sensitivity of 0.988, and a specificity of 0.380.

When chain-of-thought reasoning was incorporated, classification performance became comparatively more balanced. The few-shot+ chain-of-thought configuration achieved an AUC of 0.864 (95% CI 0.823‐0.904) and demonstrated an accuracy of 0.716, a sensitivity of 0.895, and a specificity of 0.671.

Similarly, the chain-of-thought-only configuration achieved an AUC of 0.868 (95% CI 0.830‐0.906) and demonstrated an accuracy of 0.697, a sensitivity of 0.895, and a specificity of 0.649.

Across all prompting strategies, LLM-based approaches consistently demonstrated lower discriminative performance than the outcome-trained XGBoost model.

### Model Performance at Balanced Operating Thresholds

To further evaluate threshold-dependent classification behavior beyond the predefined probability threshold of 0.5, models were additionally assessed using balanced operating thresholds derived from the Youden Index.

At the balanced threshold (*P*=.16), the XGBoost model achieved an accuracy of 0.828, a sensitivity of 0.826, and a specificity of 0.829, while maintaining an AUC of 0.913 (95% CI 0.882‐0.944; Table 2).

**Table 2.** Model performance using a balanced probability threshold.

Model	Threshold	Accuracy	Sensitivity	Specificity	AUC^a^ (95% CI)	*P* value
XGBoost^b^	0.1555	0.828	0.826	0.829	0.913 (0.882‐0.944)	—^c^
Zero-shot Learning	0.86	0.759	0.756	0.76	0.874 (0.837‐0.911)	<.001
Few-shot Learning	0.86	0.757	0.756	0.757	0.861 (0.82‐0.901)	<.001
Few-shot+ CoT^d^	0.74	0.755	0.744	0.757	0.864 (0.823‐0.904)	<.001
CoT only	0.88	0.75	0.756	0.749	0.868 (0.83‐0.906)	<.001

a AUC: area under the receiver operating characteristic curve.

b XGBoost: extreme gradient boosting.

c Not applicable.

d CoT: chain-of-thought.

For LLM-based approaches, balanced operating thresholds ranged from 0.74 to 0.88. Under these thresholds, classification performance became more symmetric across prompting strategies.

Across LLM configurations, accuracies ranged from 0.750 to 0.759, sensitivities ranged from 0.744 to 0.756, and specificities ranged from 0.749 to 0.760. Despite more balanced threshold-dependent classification behavior, differences in discriminative performance persisted, with AUC values ranging from 0.861 to 0.874 across LLM configurations.

### Explainability and Feature Importance

**Figure 1.**
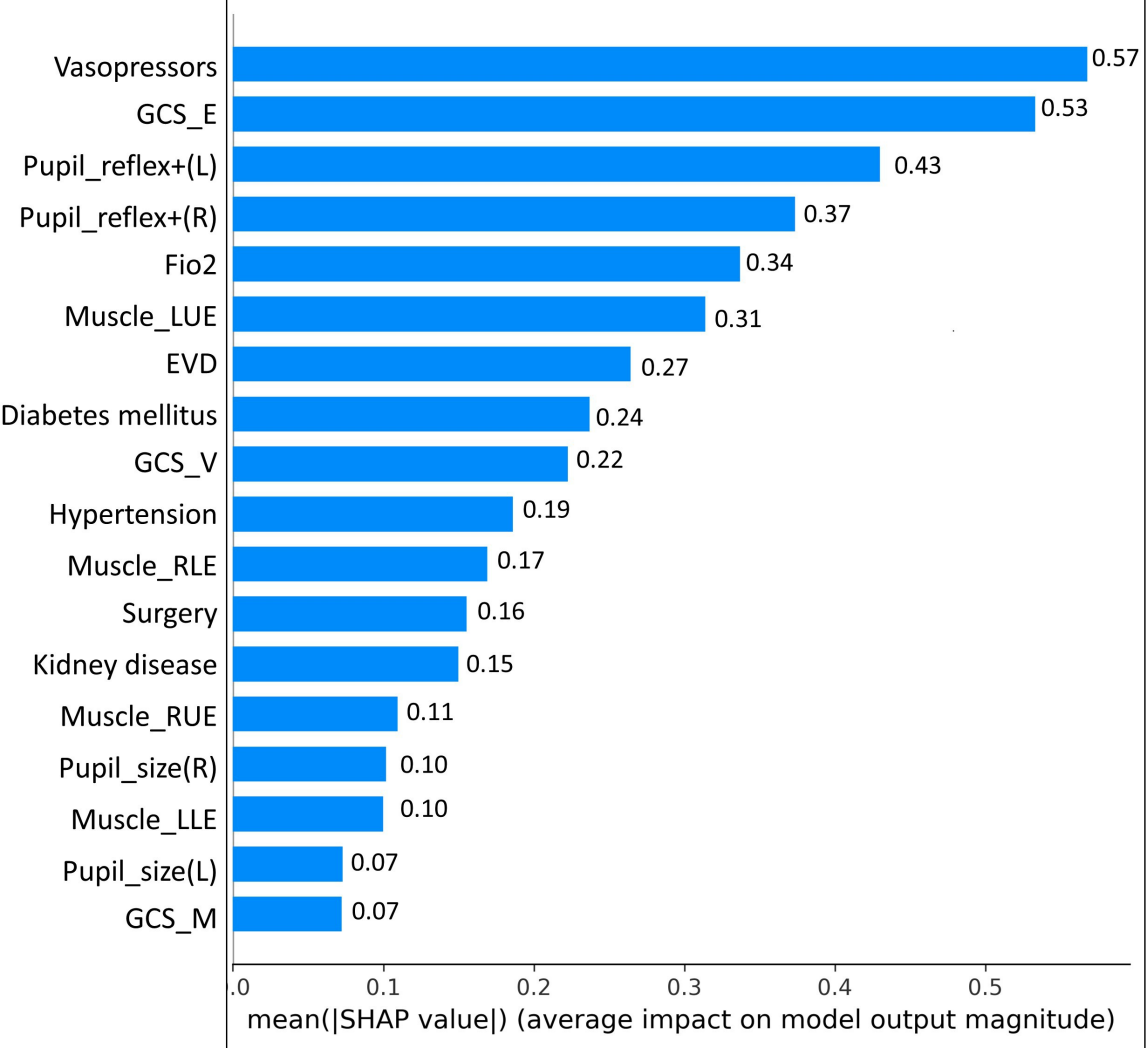
Feature importance derived from the extreme gradient boosting (XGBoost) model using Shapley Additive Explanations (SHAP). Feature importance derived from the outcome-trained XGBoost model using SHAP, demonstrating the relative contribution of 18 structured clinical variables to in-hospital mortality prediction. Features are ranked according to mean absolute SHAP values, demonstrating a hierarchical feature importance structure dominated by variables reflecting acute physiological instability and impaired neurological responsiveness. EVD: external ventricular drainage; FiO₂: fraction of inspired oxygen; GCS-E: Glasgow Coma Scale eye-opening score; GCS-M: Glasgow Coma Scale motor score; GCS-V: Glasgow Coma Scale verbal score; LLE: left lower extremity; LUE: left upper extremity; RLE: right lower extremity; RUE: right upper extremity.

Global feature importance derived from the XGBoost model using SHAP demonstrated a strongly hierarchical importance structure (Figure 1).

The most influential predictors included vasopressor use, the GCS eye-opening score, and the bilateral pupillary light reflex, which exhibited the highest mean absolute SHAP values.

In contrast, variables representing extremity muscle power and chronic comorbid conditions demonstrated comparatively lower contributions to model predictions. Notably, the GCS motor score ranked among the lowest predictors in the SHAP analysis.

### LLM-Derived Feature Prioritization

Feature prioritization generated by the LLM varied across prompting strategies but consistently emphasized neurological responsiveness variables.

**Figure 2.**
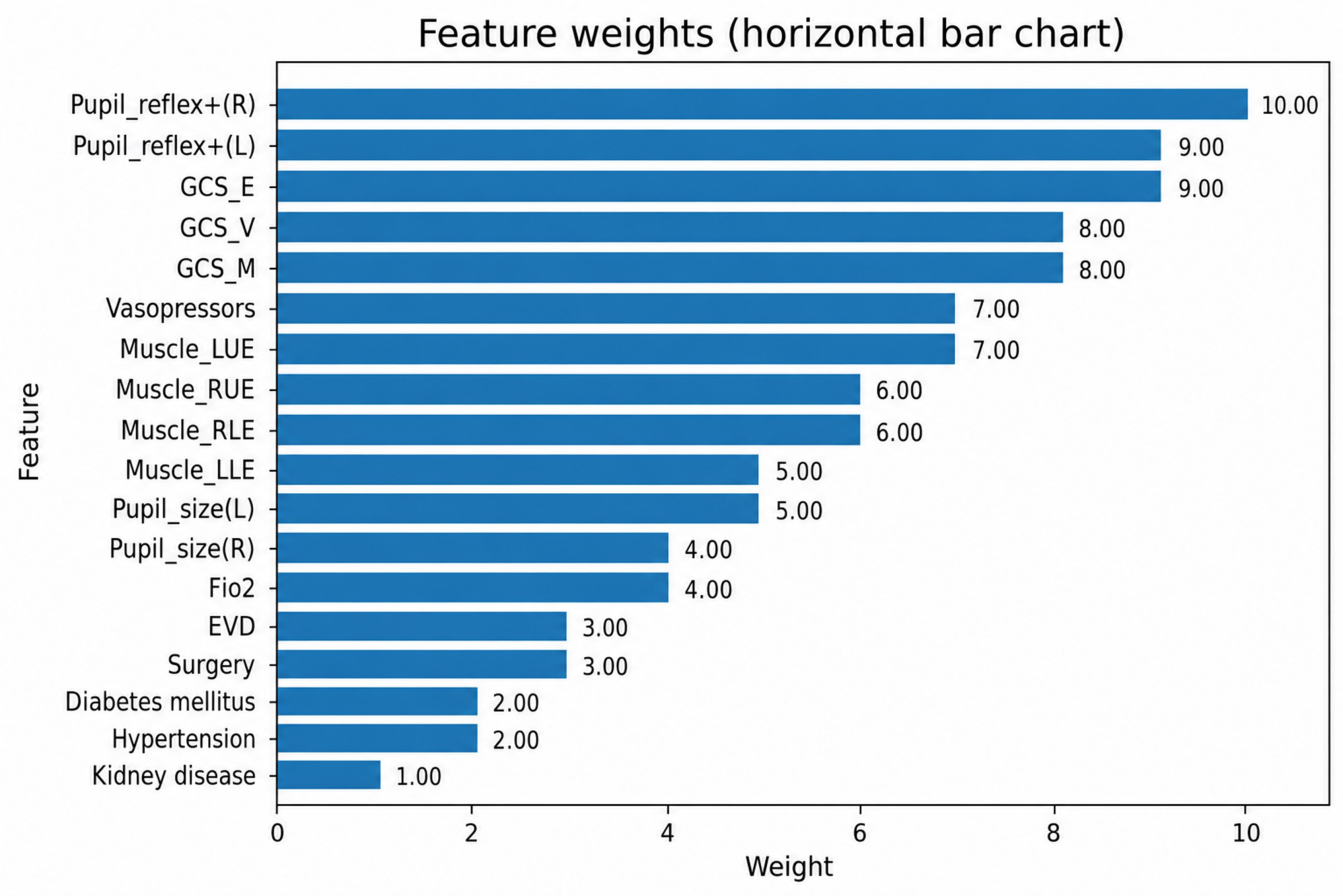
Large language model (LLM)-derived feature prioritization using zero-shot prompting. Feature prioritization was generated by the inference-only LLM under a zero-shot prompting strategy. The model was instructed to rank 18 clinical features from most to least important and assign an ordinal weight (1-10) reflecting relative importance. Features are displayed in descending order of assigned weight. These rankings represent qualitative, prompt-conditioned reasoning patterns based on generalized clinical knowledge rather than statistically derived feature attribution. EVD: external ventricular drainage; FiO₂: fraction of inspired oxygen; GCS-E: Glasgow Coma Scale eye-opening score; GCS-M: Glasgow Coma Scale motor score; GCS-V: Glasgow Coma Scale verbal score; LLE: left lower extremity; LUE: left upper extremity; RLE: right lower extremity; RUE: right upper extremity.

**Figure 3.**
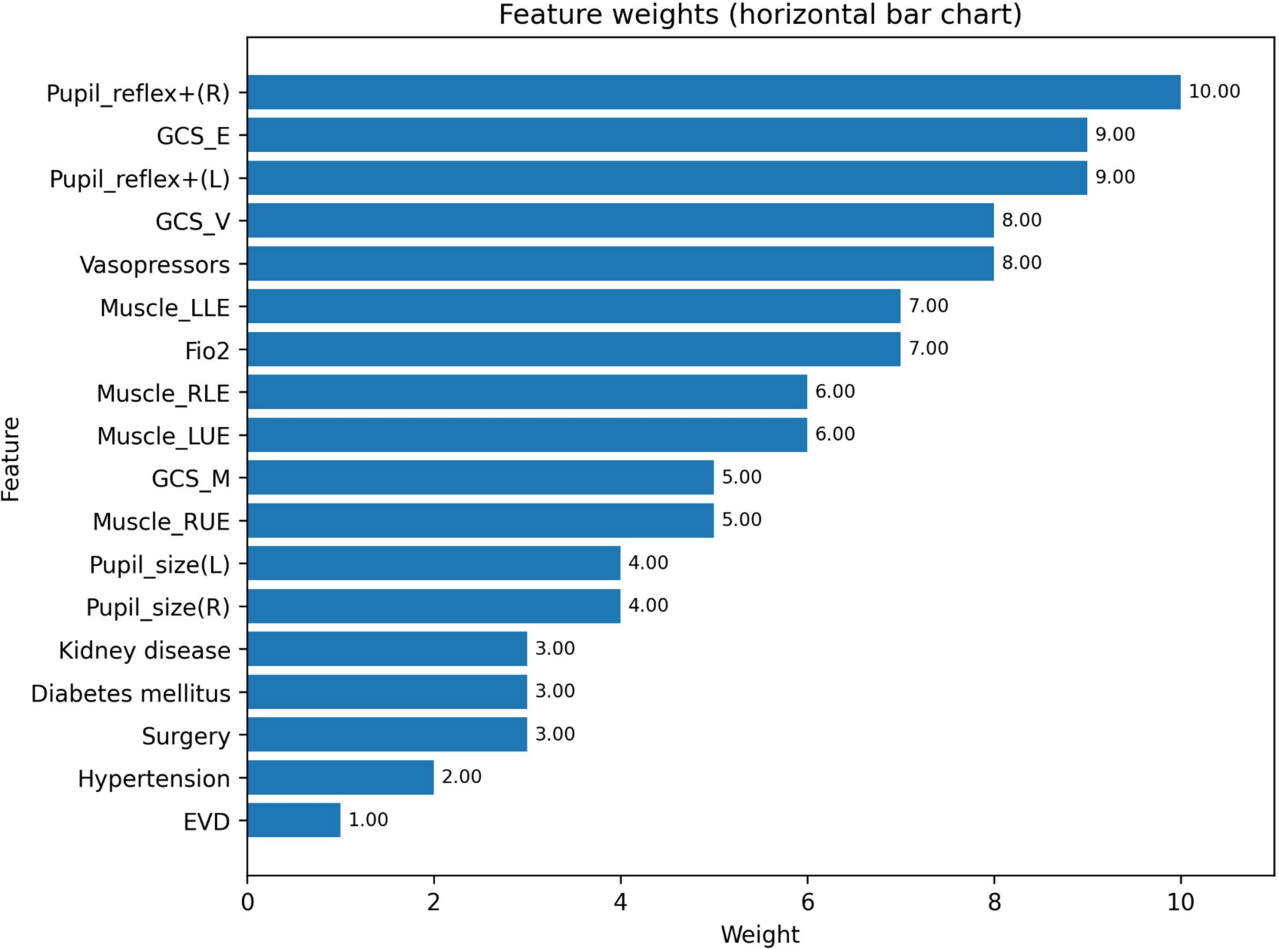
LLM-derived feature prioritization using few-shot prompting. Feature prioritization was generated by the inference-only large language model (LLM) under a few-shot prompting strategy using exemplar mortality cases from the training dataset. The model was instructed to rank 18 clinical features according to relative importance and assign ordinal weights ranging from 1 to 10. Compared with the zero-shot configuration, neurological responsiveness variables were more frequently ranked among higher-importance features, although the overall distribution of feature weights remained comparatively flatter than the hierarchical structure observed in Shapley Additive Explanations (SHAP)-derived attribution. EVD: external ventricular drainage; FiO₂: fraction of inspired oxygen; GCS-E: Glasgow Coma Scale eye-opening score; GCS-M: Glasgow Coma Scale motor score; GCS-V: Glasgow Coma Scale verbal score; LLE: left lower extremity; LUE: left upper extremity; RLE: right lower extremity; RUE: right upper extremity.

**Figure 4.**
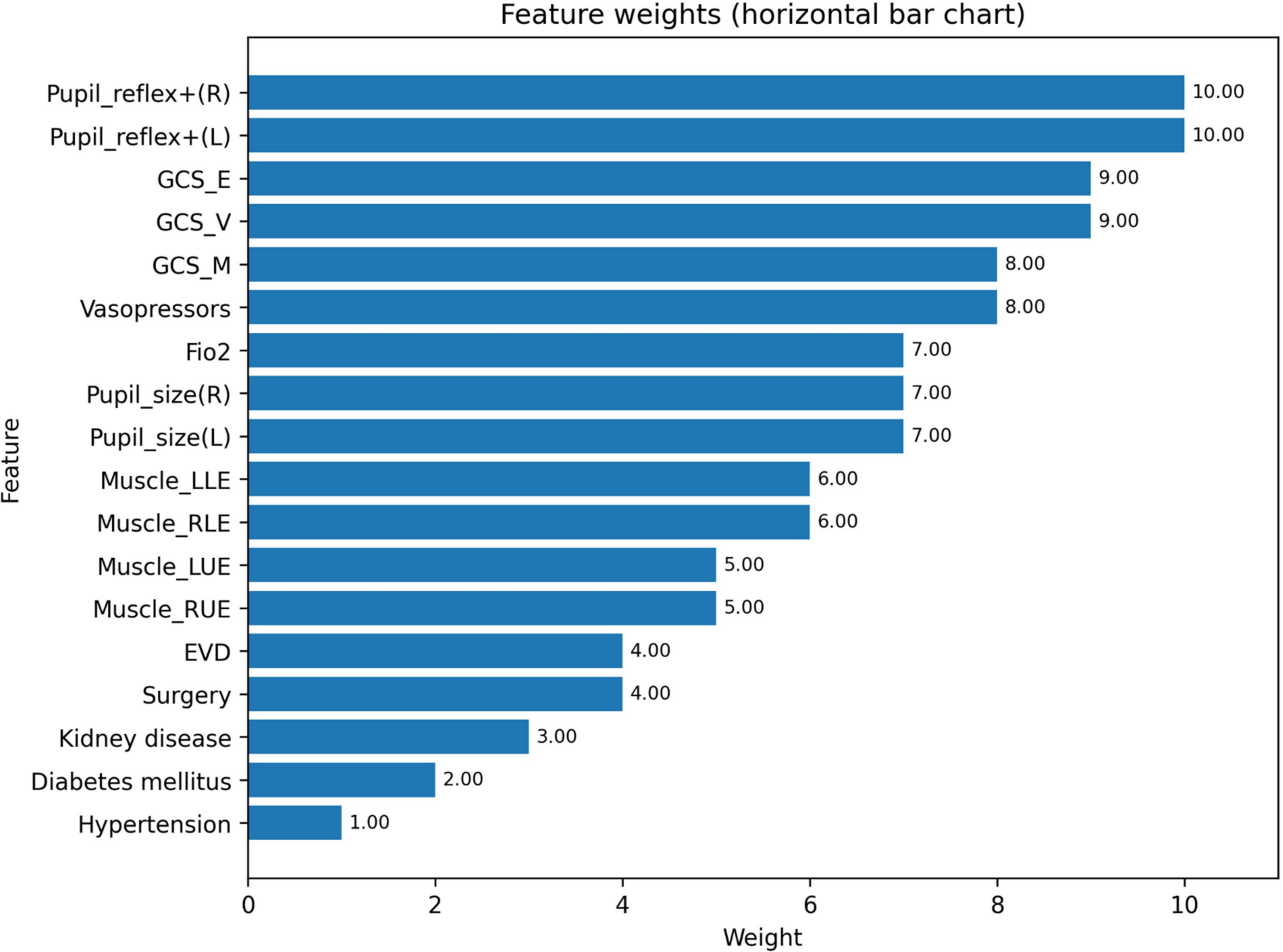
Large language model (LLM)-derived feature prioritization was conducted using few-shot prompting with chain-of-thought reasoning. Feature prioritization was generated by the inference-only LLM under a few-shot prompting strategy combined with chain-of-thought reasoning instructions. Neurological severity markers and acute intensive care variables were more frequently ranked among higher-importance features compared to other prompting configurations. The resulting feature distribution demonstrated greater separation between higher- and lower-ranked variables and showed partial similarity to the hierarchical structure observed in Shapley Additive Explanations (SHAP)-derived attributions from the extreme gradient boosting (XGBoost) model. EVD: external ventricular drainage; FiO₂: fraction of inspired oxygen; GCS-E: Glasgow Coma Scale eye-opening score; GCS-M: Glasgow Coma Scale motor score; GCS-V: Glasgow Coma Scale verbal score; LLE: left lower extremity; LUE: left upper extremity; RLE: right lower extremity; RUE: right upper extremity.

**Figure 5.**
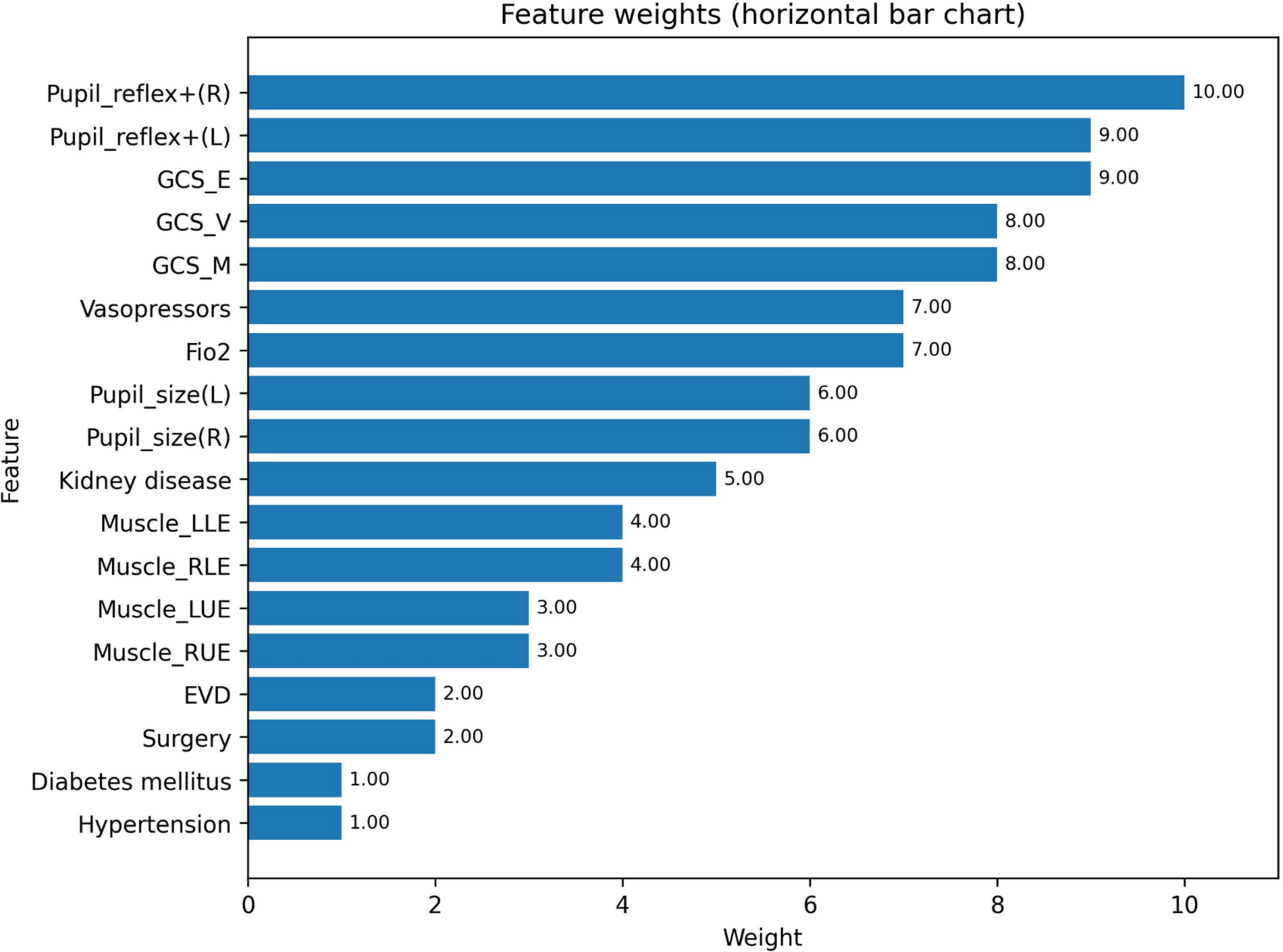
Large language model (LLM)-derived feature prioritization using chain-of-thought prompting only. Feature prioritization was generated by the inference-only LLM under the chain-of-thought–only prompting configuration without few-shot exemplar cases. Core neurological and physiological severity indicators remained among the highest-ranked variables, although the overall distribution of feature weights appeared comparatively less concentrated than that observed in the combined few-shot and chain-of-thought configuration. EVD: external ventricular drainage; FiO₂: fraction of inspired oxygen; GCS-E: Glasgow Coma Scale eye-opening score; GCS-M: Glasgow Coma Scale motor score; GCS-V: Glasgow Coma Scale verbal score; LLE: left lower extremity; LUE: left upper extremity; RLE: right lower extremity; RUE: right upper extremity.

However, the overall distribution of importance across variables was comparatively flatter than that observed in SHAP-based attribution. In particular, GCS motor score, which ranked among the lowest predictors in the SHAP analysis, was consistently ranked among higher-importance variables in LLM-generated prioritization (Figures 2-5).

### Concordance Between SHAP Attribution and LLM Feature Prioritization

To assess the relationship between statistically derived feature attribution and language-based feature prioritization, Spearman rank correlation coefficients were calculated between SHAP-derived feature importance and LLM-derived feature rankings.

Concordance between SHAP-derived feature importance and LLM-derived feature prioritization was modest across prompting strategies, with Spearman correlation coefficients ranging from 0.319 to 0.474. The highest concordance was observed in the few-shot configuration (ρ=0.474; *P*=.049), whereas correlations for the other prompting strategies did not reach statistical significance.

Overall, these findings suggest partial alignment between SHAP-derived feature attribution and LLM-derived feature prioritization patterns, although the exploratory nature and the limited statistical power of this analysis should be considered when interpreting these results (Table 3).

**Table 3.** Spearman rank correlation between Shapley Additive Explanations (SHAP)-derived feature importance and large language model (LLM)-derived feature prioritization^a^.

Prompting strategy	Spearman ρ	*P* value
Zero-shot	0.369	.13
Few-shot	0.474	.049
Few-shot + CoT^b^	0.347	.15
CoT-only	0.319	.19

a Spearman rank correlation coefficients were calculated based on the ranking of 18 clinical features. Statistical significance was defined as *P*<.05.

b CoT: chain-of-thought.

## Discussion

### Principal Findings

This study provides a controlled comparison between an outcome-trained machine learning model and an inference-only LLM using identical structured clinical inputs for mortality prediction in ICU patients with SICH.

The results demonstrate a consistent pattern: although LLM-based approaches achieved moderate discriminative performance and produced clinically plausible outputs, their operational prediction behavior differed from that of the outcome-trained model.

The XGBoost model showed comparatively stable operating characteristics across evaluated thresholds, reflecting its optimization against empirical outcome data [6,7]. In contrast, LLM-based outputs varied across prompting strategies, with substantial differences in threshold-dependent classification behavior and sensitivity-specificity trade-offs. These findings suggest that probability-like outputs generated by inference-only LLMs may vary substantially across prompting conditions and may not consistently correspond to the same operational decision behavior across those conditions.

In addition, concordance between SHAP-derived feature importance and LLM-derived prioritization was modest. While both approaches emphasized clinically relevant variables, the relative weighting and feature hierarchy differed, suggesting only partial alignment between language-based reasoning and empirically derived predictive structure [17].

Taken together, these findings support a distinction between outcome-trained predictive systems and inference-only language models. Machine learning models are explicitly optimized to align with observed outcome distributions, whereas LLMs generate outputs through knowledge-driven reasoning processes without outcome-specific optimization. As a result, LLM-generated probability-like outputs may differ from outcome-trained model predictions in their interpretation and operational behavior [11,16].

Importantly, this study reflects a practical deployment scenario in which general-purpose LLMs are applied without task-specific fine-tuning or calibration. Under such conditions, caution may be warranted when interpreting LLM-generated numerical outputs as quantitative clinical risk estimates.

### Discrimination Versus Probability Interpretation in Clinical Prediction

A key observation from this study is the distinction between discriminative performance and probability interpretation. Although LLM-based approaches achieved moderate AUC values, their threshold-dependent classification behavior varied across prompting strategies. In this study, AUC was interpreted only as a measure of discrimination and ranking ability, whereas threshold-dependent classification behavior was treated as a separate operational property rather than a direct measure of calibration.

At a fixed probability threshold, LLM predictions consistently favored high sensitivity at the expense of specificity. However, the fixed probability threshold of *P*=.5 does not represent an equivalent operating point across the evaluated models. The XGBoost model demonstrated an optimal threshold substantially below 0.5, whereas the optimal thresholds for the LLM-based approaches ranged from 0.74 to 0.88. Consequently, the sensitivity and specificity profiles observed at *P*=.5 should be interpreted as reflecting threshold-dependent classification behavior under a common reference threshold rather than performance at comparable model-specific operating conditions. The observed high-sensitivity and low-specificity pattern in the few-shot condition may also have been influenced by the composition of the exemplar set. Because only mortality cases were provided as outcome-labeled examples, the prompting context may have preferentially emphasized features associated with death, potentially contributing to the tendency toward positive classifications at a fixed probability threshold. Accordingly, the observed performance pattern should not be attributed solely to general prompt sensitivity.

When operating thresholds were adjusted using the Youden Index, threshold values differed substantially across prompting configurations. These observations suggest that LLM-generated probability-like outputs may not consistently correspond to the same underlying outcome likelihood across prompting conditions.

Because formal calibration analyses were not performed, these findings should not be interpreted as direct evidence of miscalibration. Rather, the observed variability in threshold-dependent behavior provides indirect evidence that probability outputs generated by inference-only LLMs may be sensitive to contextual prompting conditions. This distinction is important because discrimination, calibration, and threshold behavior represent related but conceptually distinct dimensions of predictive model performance [11].

These findings have practical clinical implications. In many clinical settings, management decisions rely on predefined probability thresholds. If probability outputs vary substantially across prompting conditions, threshold-based decision-making may become inconsistent, even when discriminative performance appears acceptable [16].

More broadly, these results align with prior concerns that AI systems may generate clinically plausible outputs that do not necessarily reflect empirically optimized predictive relationships [17,18]. Our findings extend this concern to structured clinical prediction tasks involving inference-only LLM deployment.

The findings should, therefore, be interpreted primarily as observations regarding operational behavior under different prompting conditions rather than as a formal evaluation of probabilistic calibration.

### Comparison of SHAP-Derived Attribution and Language-Based Feature Prioritization

The comparison between SHAP-derived attribution and LLM-derived feature prioritization further illustrates the differences between data-driven prediction and language-based reasoning.

The XGBoost model produced a strongly hierarchical feature-importance structure, dominated by variables reflecting acute physiological instability and neurological impairment, consistent with established prognostic factors in intracerebral hemorrhage [4].

In contrast, LLM-derived rankings were comparatively flatter and varied across prompting strategies. Variables with limited contribution in the SHAP analysis were sometimes assigned relatively high importance by the LLM. This pattern suggests that LLM prioritization may be influenced more strongly by generalized clinical plausibility than by dataset-specific predictive contribution.

These findings highlight important conceptual differences in how “explanation” is generated. SHAP values quantify feature contributions within an outcome-trained model using empirically observed data relationships [19,20]. In contrast, LLM-generated prioritization reflects narrative reasoning processes derived from generalized medical knowledge and prompt-dependent inference [17].

Accordingly, LLM-generated explanations should be interpreted as qualitative reasoning patterns rather than direct equivalents of statistically derived feature attribution. Although such explanations may appear clinically coherent, they may not correspond fully to the predictive structure learned from observed outcome data.

### Comparison With Prior Work

Previous studies evaluating LLMs in clinical settings have primarily focused on task performance, response quality, or benchmark accuracy [12-14]. In parallel, outcome-trained machine learning models have demonstrated strong performance in structured clinical prediction tasks using electronic health record data [7].

However, relatively little attention has been paid to how probability-like outputs generated by inference-only LLMs should be interpreted in structured prediction settings. Most prior evaluations have emphasized discrimination metrics without examining threshold-dependent behavior or the relationship between probability outputs and prompting conditions [11,16].

By directly comparing inference-only LLMs and an outcome-trained machine learning model using identical structured inputs, this study addresses this gap. The findings suggest that similar discriminative performance does not necessarily imply equivalent operational behavior across prompting conditions.

Furthermore, the modest concordance observed between SHAP-derived attribution and LLM-derived prioritization reinforces the distinction between empirically optimized prediction and language-based reasoning. While explainability frameworks quantify feature contributions using observed outcome data [19,20], LLM-generated reasoning reflects generalized medical knowledge that may not align consistently with dataset-specific predictive structure [17].

Importantly, observations regarding probability interpretation in this study are based on threshold-dependent classification behavior rather than formal calibration analyses and should therefore be interpreted as exploratory and hypothesis-generating.

### Implications for Clinical Deployment of LLMs

These findings have practical implications for the integration of LLMs into clinical workflows. Although LLM-based approaches demonstrated moderate discrimination and generated clinically plausible reasoning patterns, their outputs exhibited variability across prompting conditions.

Such characteristics may limit the use of inference-only LLMs as standalone probabilistic prediction systems in high-stakes clinical settings. When numerical outputs are interpreted as direct risk estimates, variability in threshold-dependent behavior may contribute to inconsistent decision-making.

A more appropriate role for LLMs may involve supporting clinical interpretation and contextual reasoning rather than serving as independent probability estimation systems. In contrast, outcome-trained predictive models remain important for applications requiring empirically optimized risk estimation.

From a systems perspective, these findings support a complementary framework in which LLMs and predictive models serve distinct but potentially synergistic roles. LLMs may assist with contextual interpretation and knowledge synthesis, whereas outcome-trained models provide structured prediction based on empirically observed outcome relationships.

As AI becomes increasingly integrated into clinical practice, different AI paradigms should not be assumed to be interchangeable. Evaluation frameworks should consider discrimination, probability interpretation, threshold-dependent behavior, and robustness across prompting conditions when assessing clinical AI systems.

### Limitations

Several limitations should be acknowledged. First, the study was conducted using a single retrospective ICU cohort of patients with SICH, which may limit generalizability. External validation across diverse populations is required.

Second, formal calibration analyses were not performed. Accordingly, observations regarding probability interpretation should not be taken as direct evidence of calibration performance or miscalibration. Rather, the present findings are based on observed threshold-dependent classification behavior under different prompting conditions.

Third, LLM performance may be sensitive to prompt design. Although multiple prompting strategies were evaluated, the results may not fully capture the range of possible prompting configurations or deployment environments. An additional limitation relates to the design of the few-shot prompting strategy. The exemplar set consisted exclusively of mortality cases and did not include survival examples. Consequently, comparisons between zero-shot and few-shot prompting reflect not only the presence of exemplars but also the asymmetric provision of outcome-labeled information. This design may have influenced the sensitivity and specificity characteristics observed in the few-shot condition and should be considered when interpreting cross-strategy comparisons. Although the few-shot condition incorporated outcome-labeled exemplars through in-context prompting, no parameter updating or model fine-tuning was performed. In addition, the evaluated LLM was accessed through a proprietary web-based deployment environment rather than a version-locked application programming interface framework. Exact replication of the LLM inference environment is limited because the ChatGPT web interface did not expose a checkpoint identifier, hidden model subversion, or complete inference configuration. Therefore, the LLM results should be interpreted as observations from the GPT-5.3 Instant web-interface model used during the inference period, rather than from a version-locked model.

Fourth, comparisons between SHAP-derived attribution and LLM-derived feature prioritization are inherently limited because these approaches represent different conceptual constructs. The concordance analysis was exploratory in nature and based on only 18 ranked variables, limiting statistical power. Accordingly, the observed correlations should be interpreted cautiously and not as definitive evidence of agreement or disagreement between the systems.

Furthermore, LLM-derived feature prioritization was based on single-query outputs rather than repeated sampling across independent inference runs. The analysis therefore reflects prompt-conditioned ranking behavior under a specific deployment setting rather than stable population-level attribution estimates.

An important limitation of this study is that the evaluated deployment setting differs substantially from many currently proposed clinical applications of LLM. The study focused on structured clinical prediction using tabular ICU variables and direct scalar probability estimation, whereas many clinical LLM applications emphasize natural language reasoning, narrative interpretation, dialogue-based decision support, retrieval augmentation, or multimodal information integration.

Therefore, the findings should be interpreted specifically within the context of inference-only LLM deployment for structured prediction tasks and may not generalize to specialized medical LLMs or alternative clinical deployment paradigms.

## Conclusions

This study provides a controlled comparison between an outcome-trained machine learning model and inference-only LLM approaches using identical structured clinical inputs for mortality prediction in ICU patients with SICH.

Although inference-only LLM approaches achieved moderate discriminative performance, their outputs demonstrated substantial variability across prompting strategies, including differences in threshold-dependent classification behavior and feature prioritization patterns.

These findings highlight important distinctions between outcome-trained predictive models and inference-only language models in structured clinical prediction tasks. While machine learning models are explicitly optimized using observed outcome data, inference-only LLMs generate outputs through generalized reasoning processes that are not specifically optimized for quantitative probability estimation.

**Figure 6.**
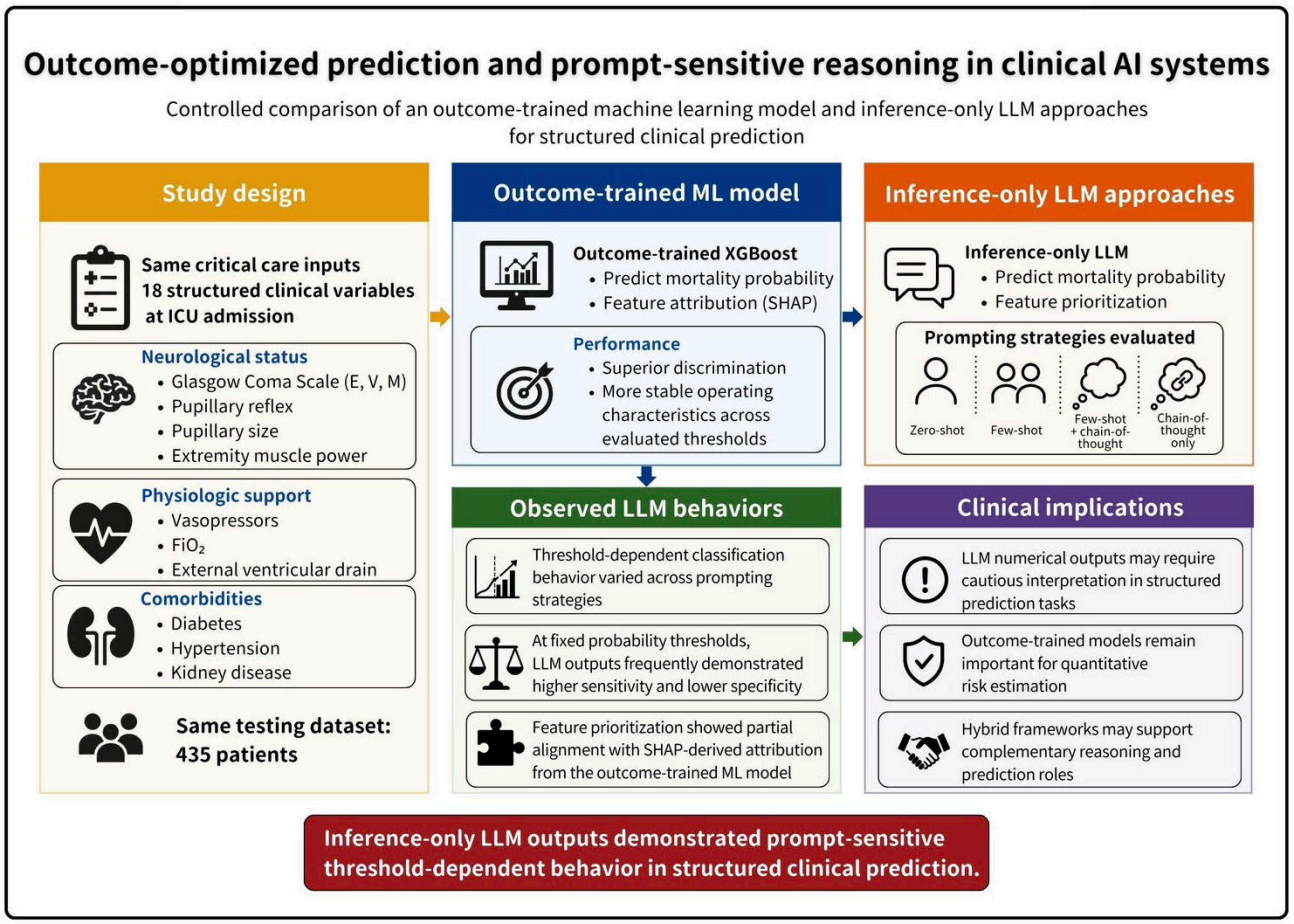
Graphical abstract of the study design, principal findings, and conceptual implications. An outcome-trained extreme gradient boosting (XGBoost) model and inference-only large language model (LLM) approaches were compared for intensive care unit (ICU) mortality prediction using identical structured clinical inputs from patients with spontaneous intracerebral hemorrhage. The outcome-trained model demonstrated superior discriminative performance and more stable operating characteristics across evaluated thresholds, whereas LLM-based approaches showed variability in threshold-dependent classification behavior across prompting strategies. Feature prioritization generated by the LLM demonstrated only partial alignment with Shapley Additive Explanations (SHAP)-derived attributions from the outcome-trained model. These findings support cautious interpretation of LLM-generated numerical outputs when applied to structured clinical prediction tasks. AI: artificial intelligence; CoT:chain-of-thought; FiO_2_: fraction of inspired oxygen; ML: machine learning.

Accordingly, caution may be warranted when interpreting LLM-generated numerical outputs as direct clinical risk estimates in the absence of task-specific optimization or calibration. A complementary framework integrating outcome-trained predictive models with LLM-assisted clinical reasoning may provide a more reliable direction for future clinical decision support systems. The overall study design, principal findings, and conceptual implications are summarized in Figure 6.

## Supplementary material

10.2196/29701 Multimedia Appendix 1 Conceptual framework illustrating methodological differences between outcome-trained machine learning models and inference-only large language models in structured clinical prediction tasks.

10.2196/29701 Multimedia Appendix 2 Large language model (LLM) prompting framework and inference instructions.

## Abbreviations

AI: artificial intelligence
AUC: area under the receiver operating characteristic curve
GCS: Glasgow Coma Scale
ICU: intensive care unit
LLM: large language model
SHAP: Shapley Additive Explanations
SICH: spontaneous intracerebral hemorrhage
XGBoost: extreme gradient boosting
