# Supplementary figures and images for: Prompt-Sensitive Decision Behavior of Large Language Models in Intensive Care Unit Mortality Prediction for Spontaneous Intracerebral Hemorrhage: Comparative Benchmarking Study
Source: J Med Internet Res. 2026 Jul 8;28:e29701. doi: 10.2196/29701 (PMC13347081; doi:10.2196/29701)

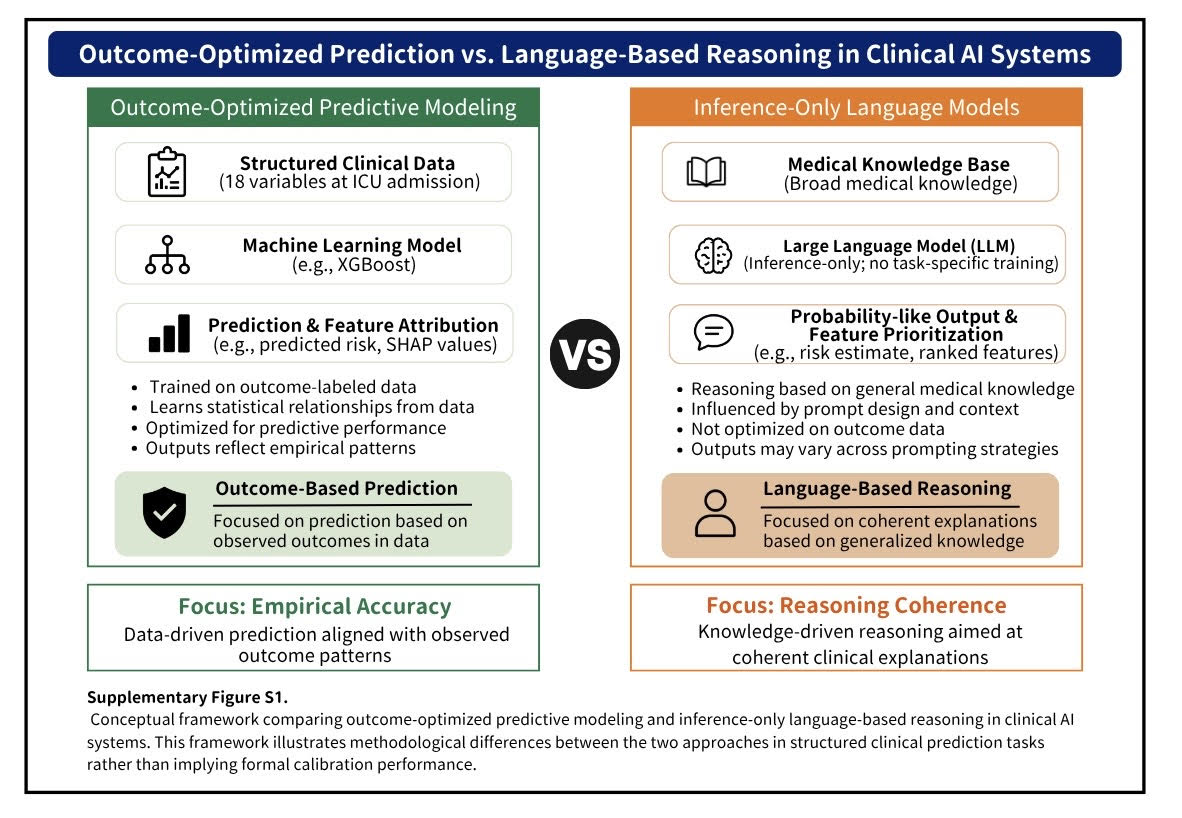

Supplement: Multimedia Appendix 1 [file jmir-v28-e29701-s001.png]
