# Supplementary material for: Prompt-Sensitive Decision Behavior of Large Language Models in Intensive Care Unit Mortality Prediction for Spontaneous Intracerebral Hemorrhage: Comparative Benchmarking Study
Source: J Med Internet Res. 2026 Jul 8;28:e29701. doi: 10.2196/29701 (PMC13347081; doi:10.2196/29701)
Supplement: Multimedia Appendix 2 [file jmir-v28-e29701-s002.docx]

**Supplementary Material 1. LLM Prompting Framework and Inference Instructions**

You are an experienced neurosurgical clinician evaluating ICU patients with spontaneous intracerebral hemorrhage (SICH). A structured dataset of patients with SICH will be provided in CSV format. Each row represents one patient and contains 18 structured clinical variables collected at ICU admission.

Based only on the provided clinical variables and your general medical knowledge, estimate the patient’s in-hospital mortality probability and record the predicted probability P (0.00–1.00) in the “AI” column.

Definition of probability output:
• P = estimated probability of in-hospital mortality
• Higher P values indicate higher estimated mortality risk

Definitions of the 18 structured clinical variables:

1. pupil_reflex+(L): Left pupil reflex
2. GCS_E: Glasgow Coma Scale – Eye opening
3. pupil_reflex+(R): Right pupil reflex
4. vasopressors: Vasopressor use
5. FiO₂: Fraction of inspired oxygen
6. GCS_V: Glasgow Coma Scale – Verbal response
7. Muscle_LUE: Muscle strength of the left upper extremity
8. GCS_M: Glasgow Coma Scale – Motor response
9. pupil_size(R): Right pupil size
10. EVD: External ventricular drain
11. Muscle_RUE: Muscle strength of the right upper extremity
12. Muscle_RLE: Muscle strength of the right lower extremity
13. Muscle_LLE: Muscle strength of the left lower extremity
14. Diabetes mellitus: Diabetes mellitus
15. kidney disease: Kidney disease
16. surgery: Surgical intervention
17. Hypertension: Hypertension
18. pupil_size(L): Left pupil size

Restrictions:
• Use only the information provided in the prompt and general medical knowledge.
• Do not access external resources or additional datasets.
• Do not modify the original input variables.

**Mode 1: Zero-shot Prompting**

• A batch of SICH patient data (CSV format) will be provided.
• Generate mortality probability predictions using only the provided feature values without exemplar reference cases.
• Output the predicted probability P for all patients.
• For the first 5 patients only, provide concise step-by-step reasoning for output verification.
• Output results in CSV format with P standardized to two decimal places.
• If the number of patients exceeds 500, process the dataset sequentially in batches of up to 500 patients.

**Mode 2: Few-shot Prompting**

A training dataset containing 199 mortality cases (Mortality = 1) will first be provided in CSV format.

• Use these exemplar mortality cases as contextual references for subsequent inference.
• A separate batch of SICH test patient data will then be provided.
• Generate mortality probability predictions using the provided feature values and exemplar contextual references.
• Output the predicted probability P for all patients.
• For the first 5 patients only, provide concise step-by-step reasoning for output verification.
• Output results in CSV format with P standardized to two decimal places.
• If the number of patients exceeds 500, process the dataset sequentially in batches of up to 500 patients.

[Upload training dataset CSV file]

**Mode 3: Few-shot Prompting with Chain-of-Thought Reasoning**

A training dataset containing 199 mortality cases (Mortality = 1) will first be provided in CSV format.

• Use these exemplar mortality cases as contextual references for subsequent inference.

Suggested reasoning framework:

1. Evaluate each clinical variable individually and identify potential high-risk findings.
2. Assess possible interactions among abnormal neurological and physiological variables.
3. Integrate clinically relevant risk indicators to estimate overall mortality probability.

• A separate batch of SICH test patient data will then be provided.
• Generate mortality probability predictions using the provided feature values and exemplar contextual references.
• For the first 5 patients only, provide concise step-by-step reasoning for output verification.
• Output results in CSV format with P standardized to two decimal places and recorded in the “AI” column.
• If the number of patients exceeds 500, process the dataset sequentially in batches of up to 500 patients.

[Upload training dataset CSV file]

**Mode 4: Chain-of-Thought Prompting Only**

Suggested reasoning framework:

1. Evaluate each clinical variable individually and identify potential high-risk findings.
2. Assess possible interactions among abnormal neurological and physiological variables.
3. Integrate clinically relevant risk indicators to estimate overall mortality probability.

• Generate mortality probability predictions without exemplar reference cases.
• Output results in CSV format with P standardized to two decimal places and recorded in the “AI” column.

The complete SICH test dataset (CSV format) will then be provided.

• Predict the mortality probability of each patient according to the selected prompting strategy.
• Preserve the original row order and all original columns.
• Add only the “AI” column containing the predicted probability P.
• Standardize probability outputs to two decimal places.
• If the number of patients exceeds 500, process sequentially in batches of up to 500 patients.

After generating predictions, rank the 18 clinical variables from most important to least important according to qualitative clinical importance and assign each variable an ordinal weight ranging from 1 to 10.
